# A new species of the genus *Amolops* (Anura: Ranidae) from high-altitude Sichuan, southwestern China, with a discussion on the taxonomic status of *Amolops kangtingensis*

**DOI:** 10.24272/j.issn.2095-8137.2017.022

**Published:** 2017-05-18

**Authors:** Liang Fei, Chang-Yuan Ye, Yu-Fan Wang, Ke Jiang

**Affiliations:** ^1^Chengdu Institute of Biology, Chinese Academy of Sciences, Chengdu Sichuan 610041, China; ^2^State Key Laboratory of Genetic Resources and Evolution, Kunming Institute of Zoology, Chinese Academy of Sciences, Kunming Yunnan 650223, China; ^3^Zhejiang Forest Resource Monitoring Center, Hangzhou Zhejiang 310020, China

**Keywords:** New species, Sichuan, Taxonomy, *Amolops xinduqiao* sp. nov., *Amolops kangtingensis*

## Abstract

A new species of the genus *Amolops* Cope, 1865 is described from Xinduqiao, Kangding, Sichuan. It was previously identified as *Amolops kangtingensis*, which is synonymized to *Amolops* mantzorum in this study. The new species, *Amolops xinduqiao* sp. nov., is distinguished from all other congeners by the following combination of characters: (1) medium body size, adult males SVL 41.2–47.5 mm (*n*=15, average 43.9 mm), adult females SVL 48.5–56.6 mm (*n*=15, average 52.5 mm); (2) head length equal to width or slightly wider than long; (3) tympanum small, but distinct; (4) vomerine teeth in two tiny rows, separated by a space about one vomerine teeth row; (5) bony projections on lower jaw absent; (6) dorsolateral folds usually absent; (7) tarsal folds or glands on tarsus absent; (8) circummarginal groove on disc of finger Ⅰ absent; (9) tibiotarsal articulation reaching nostril or beyond; (10) webs of toe IV reaching to distal articulation, other toes fully webbed to disc; and (11) vocal sac absent in males.

## INTRODUCTION

The genus *Amolops* Cope, 1865, which contains 51 species at present (Frost, 2017), is distributed from Nepal, northern India, western and southern China to Malaysia. These species inhabit rocky streams or water falls, from which they receive their common name as cascade frogs. The monophyly of this genus is supported by previous molecular studies (Cai et al., 2007; Stuart, 2008).

*Amolops kangtingensis* (Liu, 1950) was confused with *Amolops mantzorum* (David, 1871) for a long time. Liu & Hu (1961) synonymized *A. kangtingensis* with *A. mantzorum* due to the difficulty in identification between these two species under preservative condition. Based on karyotyping studies, Wu et al. (1987) supported the validity of *A. kangtingensis*, which was followed by Dubois (1992) and Zhao & Adler (1993), but rejected by Fei et al.(2005, 2009b, 2012). Lu et al. (2014) discussed the validity of *A. kangtingensis* according to molecular data, and Zhang et al. (2015) recognized *A. kangtingensis* through a combination of morphological, karyotypic, and molecular analyses of different populations of these two species. Based on the species delimitation by Zhang et al. (2015), high altitude (above 3 000 m) populations in the Yalong River Basin are considered different from the mid-high altitude (1 200–2 400 m) populations in the Dadu River Basin. However, the above research supporting the validity of *A. kangtingensis* did not examine the originally designated type locality of *A. kangtingensis*. These earlier studies treated Xinduqiao as the type locality; however, according to the original holotype description and information, the type locality of *A. kangtingensis* was limited to Yalagou of Kangding (in the Dadu River Basin) and *A. kangtingensis* should be synonymized with *A. mantzorum*. Thus, herein we describe the Xinduqiao population as a new species.

## MATERIALS AND METHODS

### Sampling

A total of 133 individuals (51 adult males and 82 adult females) of the new species were collected from four localities, including Xinduqiao (47 males and 75 females), Liuba (one male), Dongeluo (two males and one female), and Pengbuxi (one male and six females) of Kangding, Sichuan, China. Following euthanasia, five recently collected specimens (KIZ 014127–31 from Xinduqiao in 2016) were fixed in 10% formalin solution after sampling of tissues (in 95% ethanol), and transferred to 75% ethanol after fieldwork. Other specimens (collected from Xinduqiao in 1973 and 1980, Liuba and Dongeluo in 1980, and Pengbuxi in 1984) were fixed and deposited in 10% formalin solution. Holotype, allotype, and 126 paratypes were deposited in the museum of the Chengdu Institute of Biology (CIB), Chinese Academy of Sciences (CAS), with another five paratypes (KIZ 014127–31) deposited in the museum of the Kunming Institue of Zoolgy (KIZ), CAS.

### Morphological analysis

Fifteen males and 15 females from Xinduqiao were measured. All measurements were carried out with slide calipers to the nearest 0.1 mm. Morphological characters used and their measurement methods followed Fei et al. (2009a) and Jiang et al. (2016). The morphological characters and their abbreviations are listed below: SVL, snout-vent length; HL, head length (from posterior corner of mandible to tip of snout); HW, head width (at the angle of the jaw); SL, snout length; INS, internarial space; IOS, interorbital space; UEW, width of upper eyelid; ED, eye diameter (horizontal); TD, tympanum diameter (horizontal); LAHL, length of lower arm and hand; LAD, diameter of lower arm; HAL, hand length; HLL, hindlimb length; TL, tibia length; TW, tibia width; TFL, length of tarsus and foot; FL, foot length.

Morphological data of congeners were obtained from previously published literature (Liu, 1950; Fei et al., 2009b).

## RESULTS

According to Fei et al.(2005, 2009b), the morphological data support the Xinduqiao population as a new species of the *Amolops mantzorum* group based on the absence of a dorsolateral fold and the absence of a circummarginal groove on the disc of the first finger. The new species differs from all other species of the *A. mantzorum* group by having a relatively small body size; green or brown-colored dorsum, with relatively small spots, and without spines; and small, but distinct tympanum. Additionally, the molecular data support the differentiation between the high altitude (above 3 000 m) population of western Mt. Zheduo in the Yalong River Basin (including Xinduqiao) and the mid-high altitude (1 200–2 400 m) population of the Dadu River Basin (Lu et al., 2014; Zhang et al., 2015).

### *Amolops xinduqiao* Fei, Ye, Wang, and Jiang, sp. nov. (Figures 1-4)

*Staurois kangtingensis*: Liu, 1950, Fieldiana: Zool. Mem., 2: 349–353.

**Figure 1:**
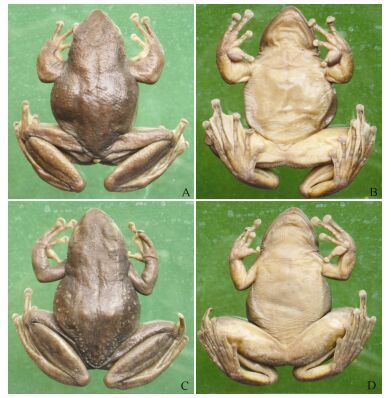
Holotype and allotype of *Amolops* *xinduqiao* sp. nov. (Photos by Liang Fei)

**Figure 2:**
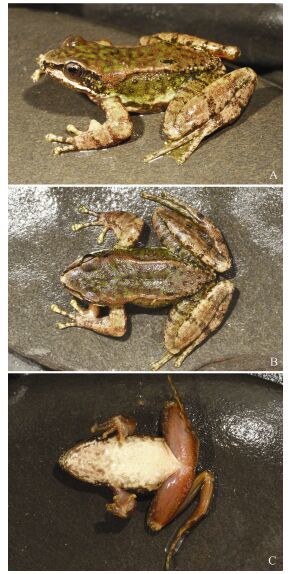
Adult male *Amolops* *xinduqiao* sp. nov. (paratype, KIZ014127) (Photos by Yufan Wang)

**Figure 3:**
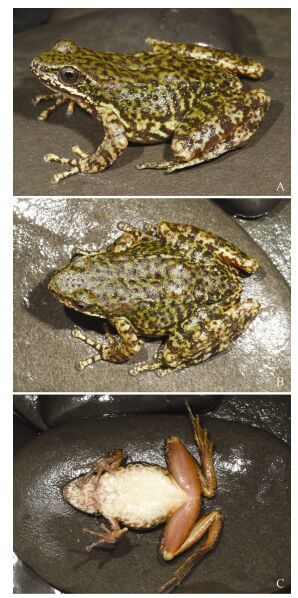
Adult female *Amolops* *xinduqiao* sp. nov. (paratype, KIZ014129) (Photos by Yufan Wang)

**Figure 4:**
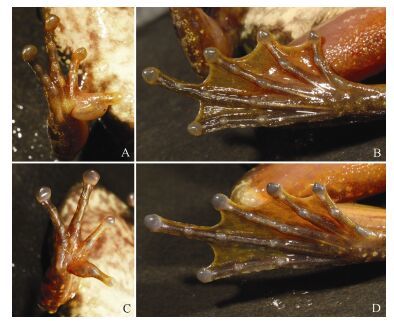
Ventral view of hand and foot of *Amolops* *xinduqiao*sp. nov.

*Amolops* *kangtingensis*:Wu, Tan, and Zhao, 1987, Acta Herpetol. Sinica, 6(4): 39–41; Lu, Bi, and Fu, 2014, Mol. Phylogenet. Evol., 73: 40–52; Zhang, Yuan, Xia, and Zeng, 2015, Sichuan J. Zool., 34(6): 801–809.

**Holotype:** CIB 80I0692 (Figure 1 A, B), adult male from Xinduqiao (新都桥, N30.14182°, E101.50044°, altitude 3 400 m), Kangding, Sichuan, PR China, collected by Yongzhao Huang on 14 June 1980.

**Allotype:** CIB 80I0696 (Figure 1 C, D), adult female, same locality and date as holotype.

**Paratypes:** Xinduqiao (same locality as holotype): 46*♂♂*, CIB 73I1151–55, 73I1167–69, 73I1173 (11 September 1973), 80I0689, 80I0704, 80I0725–26, 80I0729–30, 80I0736, 80I0738–45, 80I0747, 80I0750–55, 80I0757, 80I0760–61, 80I0765, 80I0767–73, 80I0775 (14 June 1980), 80A0217 (04 August 1980), KIZ 014127, 014130 (31 August 2016); 74♀♀, CIB73I1156–66, 73I1170–1172, 73I1174 (11 September 1973), 80I0683–88, 80I0690–91, 80I0693–95, 80I0697–0703, 80I0705–0724, 80I0727–28, 80I0731–35, 80I0737, 80I0746, 80I0748–49, 80I0759, 80I0762–64, 80I0766, 80I0774 (04 August 1980), 840735 (23 May 1984), KIZ 014128–29, 014131 (31 August 2016). Liuba (六巴), altitude 3 500 m: 1*♂*, 80I0516 (04 June 1980). Dongeluo (东俄洛), altitude 3 415 m: 2*♂♂*, 80I0798–99 (15 June 1980); 1♀, 80I0797 (15 June 1980). Pengbuxi (朋布西), altitude 3 300 m: 1*♂*, 841289 (31 May 1984); 6♀♀, 841290–93 (31 May 1984), 841424–25 (26 May 1984).

**Diagnosis:** *Amolops xinduqiao* sp. nov. is distinguished from all other congeners by the following combination of characters: (1) medium body size, adult males SVL 41.2–47.5 mm (*n*=15, average 43.9 mm), adult females SVL 48.5–56.6 mm (*n*=15, average 52.5 mm); (2) head length equal to width or slightly wider than long; (3) tympanum small, but distinct; (4) vomerine teeth in two tiny rows, separated by a space about one vomerine teeth row; (5) bony projections on lower jaw absent; (6) dorsolateral folds usually absent; (7) tarsal folds or glands on tarsus absent; (8) circummarginal groove on disc of finger Ⅰ absent; (9) tibiotarsal articulation reaching nostril or beyond; (10) webs of toe Ⅳ reaching to distal articulation, other toes fully webbed to disc; and (11) vocal sac absent in males.

**Holotype description:** Medium body size, SVL 46.4 mm, slightly compressed in vertical direction. Head length equal to width; snout projecting forward and depressed, slightly pointed at tip; nostril lateral, at middle of snout and eye; canthus rostralis distinct, slightly constricted behind nostrils; loreal region concave and oblique; eye relatively large (ED/HL=0.40); interorbital space less than width of upper eyelid (IOS/UEW=0.85); tympanum small, but distinct, slightly less than one third of eye diameter (TD/ED=0.32); vomerine teeth weakly developed, in two tiny oblique rows between choanae, separated by a space about one vomerine teeth row; tongue pyriform, deeply notched posteriorly; bony projections on lower jaw absent; and vocal sac and vocal sac opening absent.

Forearm robust. Tips of all four fingers expended into discs, disc on finger Ⅰ smallest, on finger Ⅲ largest, approximately equal to diameter of tympanum; circummarginal grooves present on tips of outer three fingers, absent on finger Ⅰ; relative finger length Ⅰ < Ⅱ < Ⅳ < Ⅲ; subarticular tubercle distinct; supernumerary tubercles at base of three outer fingers; three metacarpal tubercles, elliptical and flat; and fringe absent.

Hindlimb slender, tibiotarsal articulation reaching nostril, heels overlapping when hind limbs flexed and held perpendicular to body. All five toe tips expanded into discs, relatively smaller than finger discs; relative toe length Ⅰ < Ⅱ < Ⅲ < V < Ⅳ; fully webbed on all toes, except toe Ⅳ, which is webbed to distal articulation with fringe to base of disc; elongate, oval inner metatarsal tubercle, no outer metatarsal tubercle.

Dorsal side smooth, with sparse tubercles on lateral side of head and body, and around vent; supratympanic fold absent; dorsolateral fold absent; ventral surfaces smooth except lightly flat tubercles on basal ventral surface of thigh.

**Coloration of holotype in preservative:** Dorsal and lateral sides of head and body gray-brown, with indistinct gray spots; limbs gray-brown, with indistinct gray transverse bands. Ventral sides uniformly light brown, indistinct gray spots on throat and chest.

**Second sexual characters:** Male without vocal sac or vocal sac opening; inner side of first finger with developed velvety nuptial pad, without spine; forearm of male stronger than forearm of female, and snout-vent length of male smaller than snout-vent length of female.

**Variation:** The type series measurements are summarized in Table 1–2.

**Table 1:** Morphological measurements (mm) of adult males of A*molops* *xinduqiao* sp. nov.

Number	Status	SVL	HL	HW	SL	INS	IOS	UEW	ED	TD	LAHL	LAD	HAL	HLL	TL	TW	TFL	FL
CIB80I0692	Holotype	46.4	16.4	16.4	6.5	5.3	4.0	4.7	6.6	2.1	22.8	7.5	14.2	81.6	25.6	7.4	36.5	24.4
CIB80I0704	Paratype	47.5	15.6	16.9	6.5	5.0	4.5	4.3	6.2	2.7	24.8	6.7	14.8	90.0	28.0	7.2	39.4	27.2
CIB80I0725	Paratype	42.5	14.9	14.9	6.9	5.2	4.1	3.4	6.4	2.0	21.4	5.8	12.7	76.8	24.8	6.3	34.0	23.2
CIB80I0729	Paratype	45.3	15.6	15.9	6.4	5.0	4.3	3.8	6.5	2.5	23.2	6.4	14.4	83.2	26.8	7.6	38.2	26.6
CIB80I0730	Paratype	44.9	15.0	16.8	6.4	4.9	4.5	3.7	6.2	2.4	23.7	6.1	14.1	82.1	26.5	6.8	36.6	25.9
CIB80I0737	Paratype	41.6	14.5	14.8	5.5	5.2	3.8	3.6	6.6	2.3	21.2	6.0	12.9	70.8	23.9	5.8	32.9	22.9
CIB80I0738	Paratype	41.8	14.6	15.1	5.6	4.7	4.3	3.6	5.8	2.1	21.7	5.4	13.5	79.1	24.5	5.8	34.3	22.6
CIB80I0739	Paratype	42.1	14.5	14.8	6.4	4.9	3.8	3.3	5.8	2.4	22.2	5.7	13.6	76.9	24.6	6.8	35.3	24.7
CIB80I0740	Paratype	41.2	15.3	15.0	6.4	5.0	4.1	3.6	6.4	2.0	22.1	6.0	12.9	72.1	23.6	6.6	33.9	24.0
CIB80I0741	Paratype	41.9	14.8	14.4	6.3	4.8	3.5	3.9	6.7	2.2	21.1	5.9	12.7	72.4	24.0	5.9	33.9	22.7
CIB80I0742	Paratype	42.6	14.0	14.6	6.0	4.8	3.7	3.4	6.2	2.5	21.5	5.4	12.9	77.1	24.8	6.7	35.3	24.3
CIB80I0743	Paratype	45.0	15.6	15.9	6.6	5.3	4.4	4.0	6.3	2.1	22.3	6.6	13.1	75.2	25.4	6.4	35.6	25.6
CIB80I0744	Paratype	45.4	14.2	15.2	5.7	4.4	4.4	3.5	6.2	2.3	23.0	6.1	13.6	81.7	25.7	6.8	36.8	25.8
CIB80I0747	Paratype	45.8	15.8	16.1	5.9	5.2	3.6	3.5	6.9	2.2	22.5	6.6	13.7	77.8	25.7	7.3	36.5	25.4
CIB80I0775	Paratype	44.2	14.6	14.3	6.1	4.9	4.0	3.4	6.1	2.5	21.9	6.0	12.9	74.5	24.3	7.2	33.7	23.8
Range		41.2-	14.0-	14.3-	5.5-	4.4-	3.5-	3.3-	5.8-	2.0-2.7	21.1-	5.4-	12.7-	70.8-	23.6-	5.8-	32.9-	22.6-
		47.5	16.4	16.9	6.9	5.3	4.5	4.7	6.9		24.8	7.5	14.8	90.0	28.0	7.6	39.4	27.2
Average		43.9	15.0	15.4	6.2	5.0	4.1	3.7	6.3	2.3	22.4	6.2	13.5	78.1	25.3	6.7	35.6	24.6
Ratio to SVL			34.2%	35.1%	14.1%	11.3%	9.2%	8.5%	14.4%	5.2%	51.1%	14.1%	30.7%	177.9%	57.5%	15.3%	81.0%	56.1%

**Table 2:** Morphological measurements (mm) of adult females of A*molops* *xinduqiao* sp. nov.

Number	Status	SVL	HL	HW	SL	INS	IOS	UEW	ED	TD	LAHL	LAD	HAL	HLL	TL	TW	TFL	FL
CIB8010696	Allotype	53.7	17.4	17.8	7.8	5.7	4.8	4.5	6.7	2.1	27.8	5.6	17.0	100.2	30.9	8.2	43.9	30.8
CIB8010683	Paratype	52.8	17.2	17.5	7.3	5.8	4.5	4.3	6.7	2.1	25.9	5.3	15.6	89.5	28.8	7.4	39.4	28.4
CIB8010685	Paratype	50.5	17.0	18.1	6.9	5.6	4.6	4.3	6.2	2.2	26.3	5.2	15.6	88.3	28.8	8.2	40.1	28.9
CIB8010687	Paratype	54.1	18.4	19.6	7.7	6.0	5.2	4.6	7.3	2.6	28.2	5.7	16.4	96.7	31.0	8.0	43.9	30.5
CIB8010688	Paratype	49.5	17.3	17.1	7.3	5.5	4.7	3.8	6.2	1.8	26.0	4.8	15.8	85.5	29.0	6.2	40.4	29.0
CIB8010694	Paratype	51.9	17.3	16.7	7.1	5.7	4.7	4.3	7.2	2.3	26.4	4.7	15.6	89.5	28.3	6.0	41.6	28.5
CIB8010695	Paratype	56.6	18.1	18.9	8.2	5.9	4.9	4.3	6.4	2.7	29.1	5.4	16.6	102.6	32.0	8.4	44.2	30.0
CIB8010705	Paratype	52.7	17.2	18.1	7.9	5.3	4.5	3.9	7.1	2.2	26.0	4.9	15.5	91.2	28.6	7.6	41.4	29.1
CIB8010707	Paratype	50.9	16.6	16.6	7.3	5.3	4.6	4.2	6.9	1.8	26.2	4.9	16.1	93.6	28.5	6.9	40.9	28.9
CIB8010709	Paratype	54.3	17.2	17.6	7.9	5.3	5.0	4.1	6.6	2.2	25.7	5.7	15.1	95.0	29.7	7.1	41.4	28.8
CIB8010712	Paratype	50.6	17.1	17.2	7.4	5.3	4.6	4.3	6.1	2.3	25.9	4.9	15.8	88.2	29.4	7.1	40.2	27.0
CIB8010718	Paratype	54.6	18.1	17.8	8.0	5.4	4.7	4.5	6.8	2.4	27.1	4.8	16.1	97.6	30.7	7.4	42.9	30.2
CIB8010721	Paratype	48.5	17.0	17.0	7.1	5.4	4.2	4.8	6.4	2.0	24.8	4.8	14.9	88.3	29.4	7.1	40.4	28.9
CIB80I0723	Paratype	53.6	17.8	17.7	7.3	5.8	4.6	3.8	6.3	2.8	26.9	5.4	15.6	95.3	28.1	7.6	41.1	28.3
CIB80I0753	Paratype	53.5	17.1	17.3	7.3	5.5	4.4	4.5	6.9	1.9	25.8	5.2	15.6	86.4	28.2	6.9	39.3	28.2
Range		48.5-	16.6-	16.6-	6.9-	5.3-	4.2-	3.8-	6.1-	1.8-	24.8-	4.7-	14.9-	85.5-	28.1-	6.0-	39.3-	27.0-
		56.6	18.4	19.6	8.2	6.0	5.2	4.8	7.3	2.8	29.1	5.7	17.0	102.6	32.0	8.4	44.2	30.8
Average		52.5	17.4	17.7	7.5	5.6	4.7	4.3	6.7	2.2	26.6	5.2	15.8	92.7	29.5	7.3	41.4	29.0
Ratio to SVL			33.1%	33.7%	14.3%	10.6%	8.9%	8.2%	12.7%	4.3%	50.6%	9.8%	30.1%	176.5%	56.2%	13.9%	78.9%	55.2%

**Coloration of paratype in life (KIZ 014127, adult male) (Figure 2; Figure 4A, B):** Dorsal head and body brown, with irregular small green spots; lateral head black, edge of upper lip black, white stripe from tip of snout to anterior joint of shoulder; upper part of lateral body green, with indistinct black spots, lower part of lateral body white, with black spots and large blotches; dorsal side of limbs light brown, with black transverse bands. Ventral head and body cream-white, irregular dark gray spots on throat, chest, and sides of belly; ventral side of limbs flesh-colored, without spots; toe webs gray with yellow; iris light brown, with small black spots.

**Coloration of paratype in life (KIZ 014129, adult female) (Figure 3; Figure 4C, D):** Dorsal head and body green, with reticulate black-brown spots; lateral head green, with black spots, edge of upper lip black, white stripe from tip of snout to anterior of shoulder; upper part of lateral body green, lower part of lateral body white, both with large black blotches; dorsal side of limbs green-yellow, with distinct black transverse bands and spots. Ventral head and body cream-white, irregular gray spots on throat and chest; ventral side of limbs flesh-colored, without spots; toe webs gray with yellow; iris yellow-gray, with small black spots.

**Habitat:** This frog lives at an altitude of 3 300 to 3 500 m, close to slow-moving mountain rivers or large streams in habitats with a few tall trees and with dense shrubs and weeds. Adult frogs often perch on rocks for foraging at night, but hide under rocks and grass during the day. Male and female frogs were collected during amplexus in a river near Xinduqiao on 11 September 1973, with dissection of the female specimens showing eggs with a diameter of 2.5 mm in the fallopian tube about to be spawned. Dissection of a female specimen collected on 04 June 1980, showed the biggest eggs in the ovaries to be 1.5 mm in diameter, and not yet mature. It is reasoned that the breeding season of this frog might be around September. However, breeding habitats and egg masses have yet to be confirmed.

**Comparison:** According to Fei et al.(2005, 2009b, 2012), five species, i.e., *A. granulosus* (Liu & Hu, 1961), *A. lifanensis* (Liu, 1945), *A. loloensis* (Liu, 1950), *A. mantzorum* (David, 1871), and *A. viridimaculatus*(Jiang, 1983), exist in the *A. mantzorum* species group, which is characterized by the absence of a dorsolateral fold and circummarginal groove at the first finger.

*Amolops xinduqiao* sp. nov. differs from *A. granulosus* by having a smooth dorsal surface, without spines, and no vocal sac (*v*.*s*. spines on dorsal surface and a pair of internal subgular vocal sacs) (Fei et al., 2009b).

*Amolops xinduqiao* sp. nov. differs from *A. lifanensis*, *A. loloensis*, *A. mantzorum*, and *A. viridimaculatus* by having a smaller body size, males SVL 41.2–47.5 mm (*n*=15), females SVL 48.5–56.6 mm (*n*=15) (*v.s.* males SVL 52.0–56.0 mm (*n*=5), females SVL 61.0–79.0 mm (*n*=10) in *A. lifanensis*; males SVL 54.5–62.0 mm (*n*=19), females SVL 69.5–77.5 mm (*n*=20) in *A. loloensis*; males SVL 49.0–57.0 mm (*n*=10), females SVL 59.0–72.0 mm (*n*=10) in *A. mantzorum*; males SVL 72.7–82.3 mm (*n*=19), females SVL 83.0–94.3 mm (*n*=10) in *A. viridimaculatus*) (Fei et al., 2009b).

**Figure 5:**
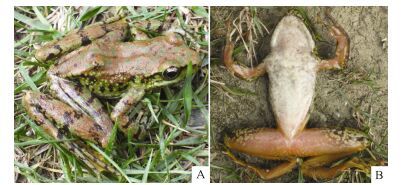
Topotype of *Amolops* *mantzorum*, Baoxing, Sichuan (Photos by Liang Fei)

*Amolops xinduqiao* sp. nov. further differs from *A. lifanensis* by having a distinct tympanum (*v.s*. tympanum indistinct); differs from *A. loloensis* and *A. viridimaculatus* by having no large brown or green spots on dorsum (*v.s*. large brown spots on dorsum in *A. loloensis*, large green spots on dorsum in *A. viridimaculatus*); differs from *A. mantzorum* by different dorsal coloration and pattern, dorsum brown, with irregular green spots, or dorsum green, with reticulate black-brown spots (*v.s.* dorsum brown, with a few large green blotches (Figure 5)) (Fei et al., 2009b).

**Etymology:**The specific name "*xinduqiao*" is named after the type locality Xinduqiao of Kangding, Sichuan. According to the Latin name, we suggest the English common name as "Xinduqiao torrent frog or Xinduqiao cascade frog", and the Chinese common name as "新都桥湍蛙".

## DISCUSSION

**Figure 6:**
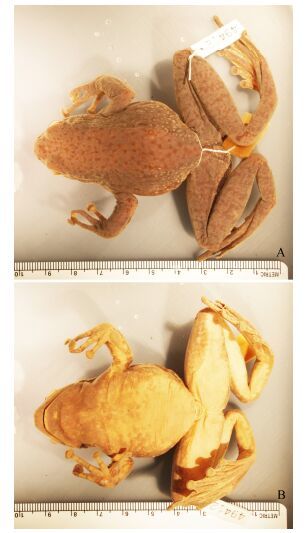
Holotype of *Amolops kangtingensis* (No. 49412, female) (Provided by Alan Resetar, the Field Museum, USA)

*Amolops kangtingensis* was described by Liu (1950). Based on the original designation, the holotype (Figure 6) is a female from "Kangting (=Kangding), Sikang (now part of Sichuan), 8 000 feet (~2 400 m)", with catalogue number 49412 in the Chicago Natural History Museum (now Field Museum of Natural History), and corresponding to field number 582 (from the collection database of the Field Museum). The holotype (No. 582) measurements were provided in the measurement table on page 351 (Liu, 1950), with body length 74.0 mm. Liu (1950) designated 29 paratypes, with the localities and altitudes provided, including: (1) 16 males, five females, and two young individuals collected with the holotype from Yalakou (=Yalagou) inside Kangting City (with three males and four females measured); four specimens from Tatu River (=Dadu River, altitude 4 500 feet (~1 370 m)) of Luting (=Luding), with the 27 frogs representing *A. kangtingensis* (=*A. mantzorum*); and (2) two paratypes from Chuwo (=Zhuwo, altitude 11 000 feet (~3 350 m)) of Luhohsien (=Luhuo) and Hsintuchiao (=Xinduqiao, altitude 11 200 feet (~3 410 m)) representing *A. xinduqiao* sp. nov.

According to the molecular data provided by Lu et al. (2014) and Zhang et al. (2015), the high altitude (above 3 000 m) populations of western Mt. Zheduo in the Yalong River Basin (including localities Jiagenba, Pengbuxi, Xinduqiao of Kangding, as well as Luhuo and Yajiang) form a monophyletic group, which should be identified as *A. xinduqiao* sp. nov. The populations of western Mt. Zheduo differ from the mid-high altitude (1200–2400 m) populations of the Dadu River Basin (including localities Kangding City (county town), Yalagou of Kangding, and Luding), which should be identified as *A. mantzorum*.

Based on the original designation, the holotype of *A. kangtingensis* was collected from Yalagou of Kangding City at an altitude of ~2 400 m, and should be identified as *A. mantzorum* according to Lu et al. (2014) and Zhang et al. (2015). The large body size (female SVL 74.0 mm) of the holotype of *A. kangtingensis* also differs from *A. xinduqiao* sp. nov. (females SVL up to 56.6 mm). Additionally, the measurements of the paratypes of *A. kangtingensis* (males SVL 53.0–57.0 mm, females SVL 70.0–74.0 mm) provided by Liu (1950) are similar to those of the topotypes of *A. mantzorum* (males SVL 49.0–57.0 mm, females SVL 59.0–72.0 mm (Fei et al., 2009b)), but different from *A. xinduqiao* sp. nov. Thus, as above, the type locality of *A*. *kangtingensis* is limited to Yalagou of Kangding, and *A*. *kangtingensis* should be synonymized with *A. mantzorum*.

## ACKNOWLEDGEMENTS

We are grateful to Dr. Jing Che (KIZ) for her kind support in this study. We thank Mr. Qi Liu for fieldwork in Xinduqiao, Prof. Yuezhao Wang, Prof. Yueying Chen, and Mr. Ke Lu (CIB) for their help and permission to examine the related museum specimens, and the Field Museum (USA) for supplying photos of holotype of *Amolops kangtingensis*. We also thank the reviewers for their critical reading of this manuscript.
